# Faith Cure and the Law

**Published:** 1901-08

**Authors:** 


### Faith Cure and the Law.

An article in the *New York Medical Record,* of April 20th, by Dr. John B. Huber, of New York, deals with "Faith Cures and the Law." In discussing clairvoyance, the doctrines of so-called "Christian Scientists" and the like, Dr. Huber says: "One would suppose now, at the beginning of the twentieth century, when men generally think sanely and reasonably, when we have come to substitute for superstition the habit of accounting for physical manifestations upon scientific bases, when science has made evident the value of sanitation, hygiene and clean living in inducing happiness and well-being and in dispelling disease, that these peculiar movements would be impossible. As a matter of fact, it is difficult to conceive how they could have ever been more numerous than they are nowadays. A great many of them have emanated from or near Boston, a region resourceful in the production of cults which have for their basic principle dissatisfaction with the universal scheme. Such movements are to be looked upon, it seems to me, as epidemic psychoses. They resemble in all essentials the innumerable epidemics of abnormal mentality which have been born, have lived, and have died during the centuries. Whatever differences there may be, are of degree rather than of kind."

The writer has investigated carefully a number of the reported Christian Science cures and he believes most of the cases to be hysterical in character. As for the claims that Christian Science is a religious system and that such healing of the sick is a part of the system they therefore cannot be compelled to desist from their work, he says: "I ask the gentlemen of the law if there is not a sharp distinction between religious liberty and license to commit in the name of religion unlawful acts. Surely, a man would not be justified in killing his child in obedience to a fanatical belief, as Abraham was about to do. But Christian Science has sacrificed the lives of many little children upon the altar of its pseudo-religion. Had not these children rights which ought to have been safeguarded?

If the Christian Scientists' position be admitted, a thug might, I submit, upon the same principle, be justified in committing murder on the ground that murder is a practice required by his religion; and a Mormon might, on the same basis, practice polygamy. And when a "healer" treats for hire a sufferer from typhoid fever, is he acting in a religious capacity? And when a person, ignorant of the most rudimentary principles of sanitary science, treats those who are sick of contagious disease, in willful disregard of laws enacted to conserve the general health of the community, may he claim respect for his personal liberty, to the jeopardy of the lives of his reasonable and sane neighbors? But from my study I derive the opinion that the assumption of religious motives by Mrs. Eddy and her associates is as false as can possibly be conceived."

The author believes many of Mrs. Eddy's followers are unquestionably good and worthy people, but he says this simply demonstrates anew the hoary and fundamental fact that mankind in large proportion evinces a burning eagerness to be gulled.

